# Overcoming adversity through diversity: aquatic carbon concentrating mechanisms

**DOI:** 10.1093/jxb/erx278

**Published:** 2017-09-05

**Authors:** Howard Griffiths, Moritz T Meyer, Rosalind E M Rickaby

**Affiliations:** 1 Department of Plant Sciences, University of Cambridge, Cambridge, UK; 2 Department of Molecular Biology, Princeton University, Princeton, NJ; 3 University of Oxford, Department of Earth Science, Oxford, UK

**Keywords:** Carbon-concentrating mechanism (CCM), convergent evolution, inorganic carbon accumulation, macrophytes, marine and freshwater, phytoplankton

**Carbon concentrating mechanism (CCM) systems, associated with evolutionarily diverse aquatic photosynthetic organisms, make a major contribution to global net primary productivity and marine carbon sequestration. Here, an overview of these global contributions is presented from their evolutionary origins, including a possible trigger for their diversification when the aqueous O_2_/CO_2_ ratio rose above parity, and a re-definition of the paradox of phytoplankton. The reviews and research in the special issue also include molecular physiology and ecology of CCMs, through to future potential applications for sustaining carbon sequestration and supporting terrestrial crop productivity.**

The inorganic carbon substrate supply needed for photosynthesis in the aquatic milieu is limited by inorganic carbon solubility and diffusion across the boundary layer, cell wall and multiple membranes to the primary carboxylase Rubisco. Various biophysical carbon concentrating mechanism (CCM) systems are found in many aquatic phytoplankters and have overcome these chemical and physical limitations. Such CCMs deliver the appropriate inorganic carbon species demanded by Rubisco (CO_2_), at an enhanced concentration which compensates for the enzyme’s low substrate affinity and competitive inhibition from oxygen.

Despite this adversity, aquatic organisms clearly punch above their weight of biomass relative to terrestrial plants. The instantaneous standing biomass crop of aquatic plants (primarily microorganisms) is 3 PgC (i.e. 10^15^ g carbon) relative to the 610 PgC usually quoted for terrestrial plant above-ground biomass. The paradox of how phytoplankton deliver an annual net primary productivity of 47.5 PgC, relative to the 56.4 PgC of their terrestrial counterparts (Field *et al.*, 1998), has long intrigued researchers. In addition, the oceanic sink for net carbon sequestration is equal to that of land plants (2.3 PgC per year), such that marine organisms also facilitate the absorption of over 25% of annual anthropogenic CO_2_ emissions (Pan *et al.*, 2011).

The original paradox of the phytoplankton was thought to reflect phylogenetic diversity in competition for limiting light and inorganic resources. The high net primary productivity, identified by Field *et al.* (1998), could be explained by the interaction between ecological and environmental factors across space and time to prevent the dominance of any one phytoplankton group. Despite this contention, it is with some amusement we note that each authority tends to claim pre-eminence for the contribution made by their particular phytoplankton clade to net primary productivity!

However, the past few decades have seen several historical paradigms overturned – such as photosynthetic acclimation to light increasing the depth of the photic zone (Richardson *et al.*, 1983; Raven *et al.*, 2017), the breadth of productivity across oceanic gyres (Johnson *et al.*, 2006; Partensky and Garczarek, 2010), and the molecular basis of niche differentiation found within cyanobacterial and eukarotic picoplankton populations in coastal and equatorial waters (Not *et al.*, 2012; Biller *et al.*, 2015). Additionally, we now recognize that more than 80% of marine primary productivity will be facilitated by some form of CCM (Raven and Beardall, 2016; Raven *et al.*, 2017).

This special issue provides a comprehensive update on aquatic carbon concentrating mechanisms, as well as reflection on how the field has progressed since the 1980s (Kaplan, 2017) together with the latest new research (see Box 1).

**Box 1. Pioneering contributions over 40 years**

The diversity of papers presented in this special issue reflects the range of contributions made at CCM9 in 2016, the ninth International Symposium on Inorganic Carbon Uptake by Aquatic Photosynthetic Organisms (Cambridge, UK; a satellite meeting following the 17th International Congress on Photosynthesis in Maastricht, The Netherlands). At the meeting, we were able to celebrate pioneering contributions over the past 40 years in person with Joe Berry, Aaron Kaplan and John Raven, and also recognize the outstanding technical and theoretical innovations made throughout this period by Murray Badger. A series of special publications has historically accompanied previous CCM Symposia, starting with the pioneering ASPP (American Society of Plant Physiologists) ‘Green Book’ proceedings from the first meeting in Asilomar (CA, USA) (Lucas and Berry, 1985), and through to that summarized by Moroney and Wee (2014). In the current special issue, we capture this progression with the highly personalized account by Aaron Kaplan of CCM research developments during those early years (Kaplan, 2017).

## Palaeohistorical and environmental drivers for CCM origins and diversity

We have more certainty about the timeline for the diversification of prokaryotic and eukaryotic clades, and their contrasting endosymbiotic exchanges, than for the origins and diversity of CCMs. Using projections based on the current Rubisco content and kinetic properties of extant cyanobacteria, Raven *et al.* (2017) suggest that the atmospheric CO_2_ concentrations ranging from ×10 current to ×4.5 current, between 1.6 to 0.6 Ga (i.e. 10^9^ years ago), could have been associated with CCM activity (see also Riding, 2006). Raven *et al.* (2017) also speculate that were *Gloeobacter* to be representative of a basal cyanobacterium, as accorded by some phylogenetic studies, then the CCM could even extend back to the Great Oxidation Event at 2.4 Ga.

Eukaryotic CCMs are generally thought to be homoplastic, with independent origins in each lineage (Raven *et al.*, 2017). The primary endosymbiosis which led to the earliest oxygenic eukaryotes was likely to have been 1.0–1.6 Ga, with green, red and glaucophyte lineages subsequently diversifying via secondary and tertiary endosymbioses (Leliaert *et al.*, 2012). The green algal lineage leading to the Chlorophyta is thought to have diverged via the Prasinophyceae from the Streptophyta by some 0.5–0.75 Ga. The red lineage (Rhodophyta) gave rise to Chromist algae (e.g. Haptophyta, Cryptophyta and Dinophyta), with haptophytes diversifying around 0.5 Ga and diatoms around 0.2 Ga (Heureux *et al.*, 2017; Young and Hopkinson, 2017). Whilst the Rubisco large subunit has provided key phylogenetic insights for this progression (Badger and Price, 2003; Price *et al.*, 2013), the co-evolution of Rubisco variants, their kinetic properties and responsiveness to CO_2_ and O_2_ remain a critical element in CCM evolution.

**Fig. 1.**
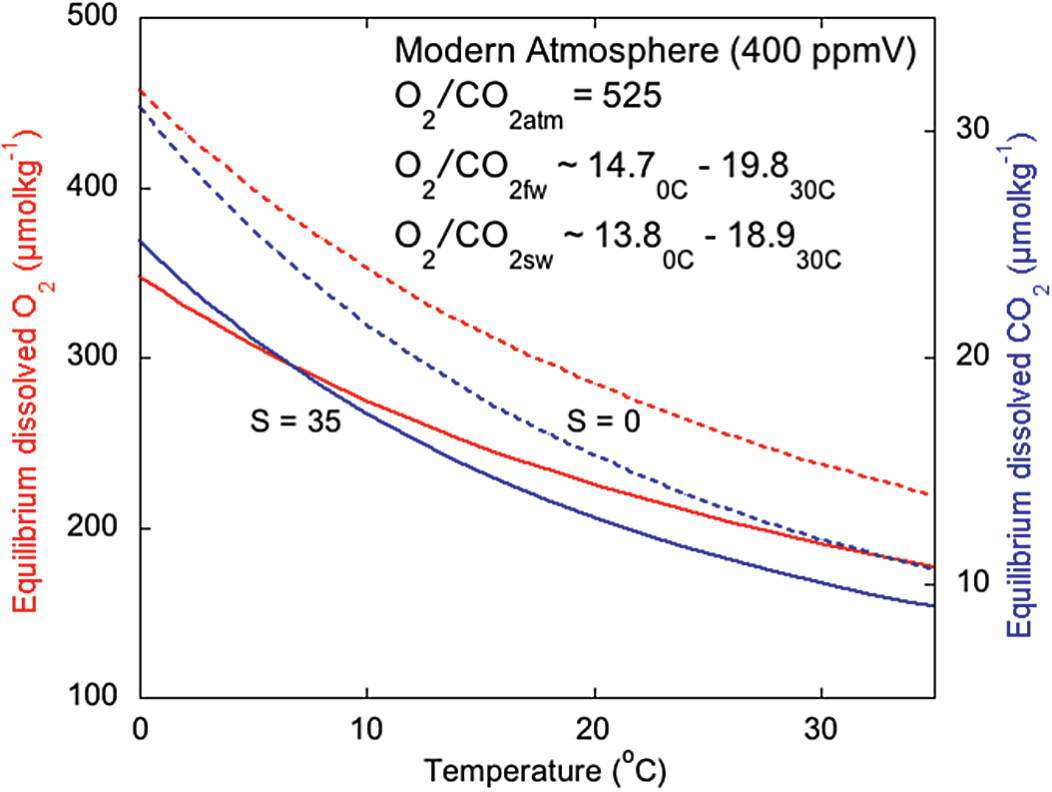
(a) The sensitivity of equilibrium dissolved CO_2_ (blue; Weiss, 1974) and O_2_ (red; Benson and Krause, 1984) concentrations to temperature and salinity (S; 0, dashed line, and 35 ppt, solid line) and the modern range (with an atmosphere of 400 ppmV) of dissolved O_2_/CO_2_ ratios for freshwater (fw) and seawater (sw) between 0 and 30 °C.

Rubisco is not only sensitive to CO_2_, rather the CO_2_/O_2_ ratio at its active site, and there is some evidence that oxygen exclusion may be an important component in many biophysical CCM systems (Meyer and Griffiths, 2013; Heureux *et al.*, 2017; Meyer *et al.*, 2017). Against the backdrop of generally declining CO_2_ and increasing O_2_ over geological history, it is an interesting thought-experiment to explore at what point rising aqueous O_2_ overtook CO_2_ to become more dominant in seawater. It is worth noting that such a chemical event would occur at different times in the atmosphere and ocean. CO_2_ is approximately 30 times more soluble than O_2_ in seawater (Fig. 1), so atmospheric CO_2_ can be 30 times less concentrated than O_2_ in the atmosphere, but the two species will be equimolar in the ocean.

**Fig. 2.**
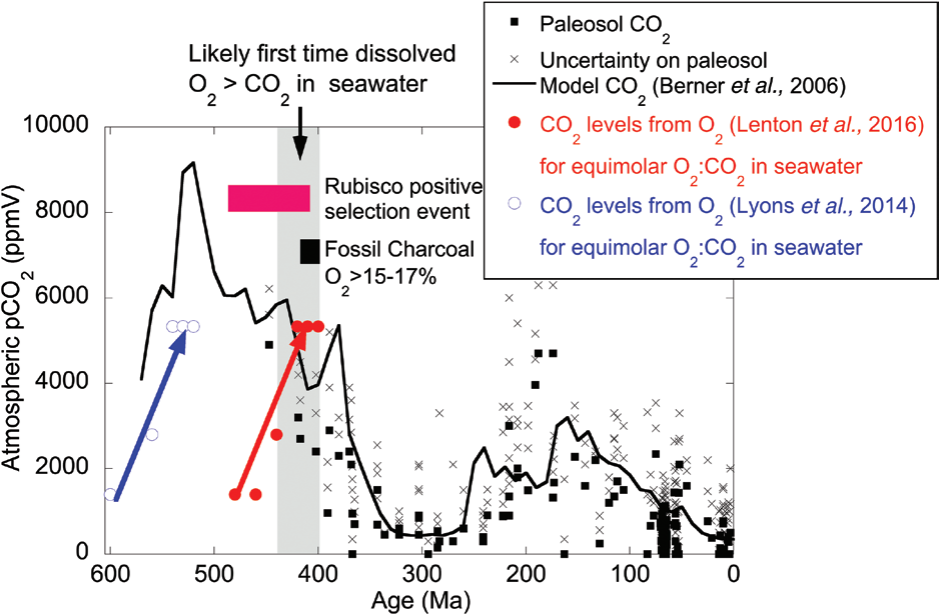
An estimate of the threshold when the concentration of dissolved aqueous O_2_ rose to a higher concentration than CO_2_ in seawater as a trigger for the emergence of CCMs (grey bar). The CO_2_ concentrations that would be equimolar with O_2_ reconstructions (blue open circles – Lyons *et al.*, 2014; red closed circles – Lenton *et al.*, 2016) are based on an approximate 30-fold difference in solubilities. The Phanerozoic history of atmospheric CO_2_ is compiled from paleosols (black squares with crosses to show the uncertainty; Royer 2006) and the GEOCARB III model (solid black line; Berner and Kothavala, 2001). Also shown are the first appearance of fossil charcoal evidence that O_2_>15–17% (black bar, Glasspool *et al.*, 2004), and a Rubisco positive selection event (magenta bar, Young *et al.*, 2012).

In the early stages of the oxygenation of the atmosphere during the Great Oxidation Event (2.4 Ga), oxygen is estimated to have risen to between 10^–2^ and 10^–1^ PAL (present atmospheric level), i.e. between 210 ppmV and 2100 ppmV (Lyons *et al.*, 2014). The best estimates of CO_2_ at this time suggest that it was probably in excess of 100 PAL (i.e. 35 000 ppmV) (Young *et al.*, 2012), so orders of magnitude more abundant than O_2_. Even if oxygen levels persisted at 0.1 to 0.2 PAL (21 000 ppmV) through the ‘boring billion’ (approximately 2 to 1 Ga) until close to the Precambrian/Cambrian boundary, CO_2_ levels would need to be 2–4 PAL (i.e. 7–1400 ppmV) to be equimolar with oxygen in the ocean, much lower than current estimates for this time. The timing then of parity between O_2_ and CO_2_ concentrations in the oceans is determined by the point at which O_2_ rose from around 0.2 PAL to close to modern values, and CO_2_ was sufficiently low to be equimolar. The most recent estimates of this O_2_ rise through 0.5 PAL (10.5%), around 450 Ma (Lenton *et al.*, 2016), requires an atmospheric concentration of 10 PAL CO_2_ (3500 ppm) to provide equimolar dissolved CO_2_ and O_2_ in marine waters, a CO_2_ level which is well within range of coincident atmospheric estimates (Fig. 2). Therefore, the environmental threshold of O_2_ overtaking CO_2_ in surface waters, driving marine organisms to provide a mechanism to boost the CO_2_/O_2_ ratio at the site of Rubisco, is likely to date to the invasion of land by the earliest plants around the late Silurian/early Devonian. Such a timing seems to agree well with an analysis of a limited number of Rubisco large subunit sequences which also finds a number of events of positive selection up to 410 Ma. This indicates that emergence of CCMs around this time indeed left a footprint in the Rubisco protein (Young *et al.*, 2012). Other, more conservative estimates, put the origins of cyanobacterial and eukaryotic CCMs following the ‘Devonian Drop’ and during the Carboniferous (Badger and Price, 2003).

There appears to be an evolutionary progression of a higher Rubisco specificity factor (selectivity for CO_2_ over O_2_) from cyanobacterial, chlorophyte and then to higher plant Form 1B Rubisco (Meyer and Griffiths, 2013). Considering the Form 1D in the marine algae, and Rhodophyta as the endo symbiont, the evolutionary trend is towards decreased Rubisco specificity factor and lower carbon affinity (higher *K*_*c*_). This has been interpreted as an evolutionary response to improved CCM activity that allows a relaxation of substrate affinity and faster catalytic turnover of the enzyme (Tcherkez *et al.*, 2006) in rhodophytes Young *et al.*, 2012; Heureux *et al.*, 2017) as well as in cyanobacteria and chlorophytes (Meyer and Griffiths, 2013). An apparent breakdown in the canonical trade-off between affinity for carbon and turnover in modern diatom Form 1D Rubisco (Young *et al.*, 2016) challenges our understanding of this evolutionary progression to lower carbon affinities. It may be necessary to consider the impact of other Rubisco catalytic parameters on Rubisco performance.

**Fig. 3.**
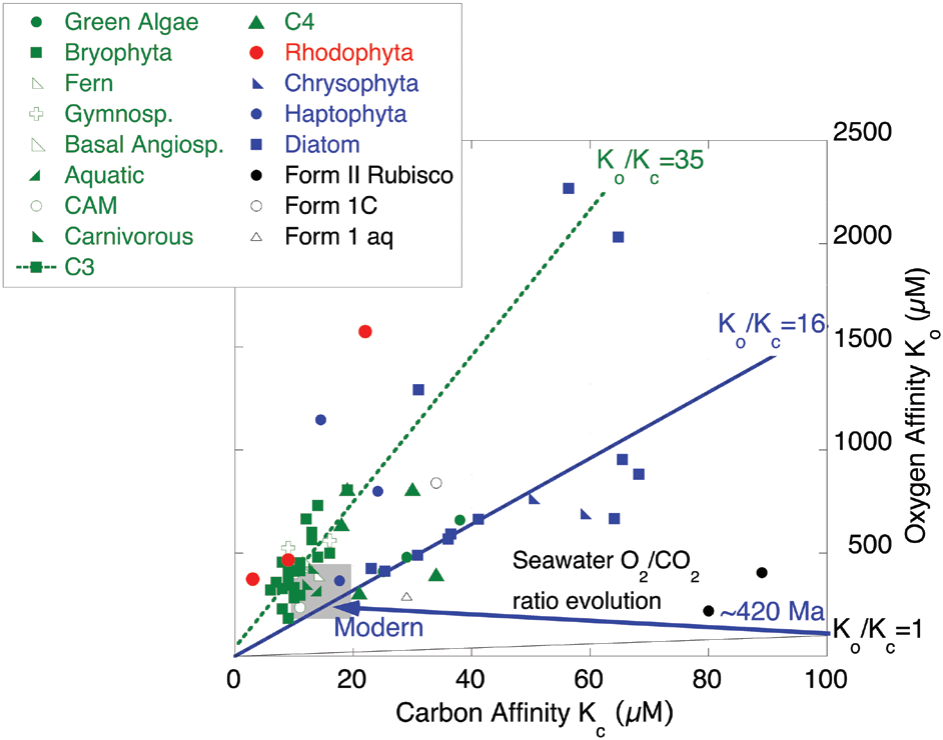
Compiled Rubisco *K*_c_ (µmol kg^–1^) versus *K*_o_ (µmol kg^–1^) (Galmés *et al.,* 2014; Young *et al.*, 2016; Heureux *et al.*, 2017) for a range of plant and algal species with Form 1B Rubisco-containing organisms in green, and Form 1D Rubisco-containing organisms in red (rhodophyta) and blue (diatoms, chrysophytes and haptophytes). Lines are plotted that indicate a *K*_c_/*K*_o_ ratio of 1 (black), 16 (blue, equivalent to modern dissolved O_2_/CO_2_ ratio of seawater) and 35 (an apparent line of best fit to the C_3_ data). The range of environmental aqueous concentrations is indicated by the grey box. The evolution of the dissolved concentrations and O_2_/CO_2_ ratio from ~1 at 420 Ma (see Fig. 2) to 16 today (‘modern’) is indicated by the blue arrow. Note that the O_2_:CO_2_ dissolved ratio of natural waters at glacial maxima of the Pleistocene was ~35.

As well as the correlations between Rubisco affinity and turnover rate for both substrates, O_2_ and CO_2_ (Heureux *et al.*, 2017), the *K*_c_ and *K*_o_ of many Rubiscos also appear to be linked (Fig. 3). The trade-off of a relaxed affinity for oxygen (higher *K*_o_), which accompanies a more efficient CCM and a higher *K*_*c*_, could improve Rubisco performance in an increasingly oxygenated environment. Whether this relationship between *K*_c_ and *K*_o_ is a constraint imposed by the structure of the enzyme (Savir *et al.*, 2010), or a response to the O_2_/CO_2_ ratio at the active site of the Rubisco remains an open question. What is curious is that the ratio of the affinities of Rubisco for O_2_ and CO_2_ (*K*_o_*/K*_c_ = ~16) of many Form 1D-containing marine algal Rubiscos, including most diatoms, and the Form 1B-containing green algae (e.g. *Chlamydomonas*), appears to match the modern dissolved O_2_/CO_2_ ratio of natural waters at equilibrium with an atmosphere containing 400 ppmV CO_2_ (16 at a representative sea surface temperature of 12 °C; see Fig. 3, and Heureux *et al.*, 2017). The affinity of these Rubiscos for O_2_ is 16 times lower than that for CO_2_. This implies that the activity of a CCM to elevate the Rubisco *K*_c_ values above the environmental availability of CO_2_ also compensates perfectly for the environmental excess of O_2_ over CO_2_. Yet there is a bimodality to the Rubisco *K*_o_*/K*_c_ ratio. The C_3_ plants and some C_4_ plants tend to fall on a line with a *K*_o_*/K*_c_ gradient of 35. Such a ratio is much higher than the dissolved O_2_/CO_2_ ratio of any modern natural waters (see Fig. 1). However, during Pleistocene glacial periods, atmospheric CO_2_ fell to ~ 180 ppmV (Lüthi *et al.*, 2008), but atmospheric O_2_ remained constant so the environmental dissolved O_2_/CO_2_ ratio was more than doubled relative to the modern (i.e. ~35). Modern Rubiscos in plants and algae, therefore, appear to be tuned in terms of O_2_/CO_2_ affinities to compensate for either glacial or modern O_2_/CO_2_ such that the Rubisco experiences a 1:1 competition between O_2_ and CO_2_ at the active site. This underpins the concept that it was the rising of environmental dissolved O_2_/CO_2_ above 1 that triggered the emergence of a CCM. It further demonstrates that the Rubisco enzyme, traditionally thought to be an inefficient relic of ancient environments, is highly dynamic. Rubisco appears to evolve its kinetics in response to environmental change over timescales of at least tens of kyrs, if not hundreds of years. The *K*_*o*_*/K*_*c*_ data appear better tuned to anthropogenic conditions rather than average interglacial atmospheric compositions. At any event, we urgently need additional analyses of Rubisco kinetic properties and sequence specificity for Chromists (Young *et al.*, 2016, 2017) as well as across the green algal lineages (Goudet, 2016) to fully understand the evolutionary history, rate of change and current diversity of carbon handling across the photosynthesizers.

A final consideration is that the origin and maintenance of CCMs might reflect environmental limitations, in addition to external inorganic carbon supply. Interactions between nitrogen availability may relate to a reduced requirement for catalytic protein following CCM induction in *Chlorella* (Beardall *et al.*, 1982), although other evidence is equivocal (Ruan *et al.*, 2017). Consistent with the earlier observation, the proportion of Rubisco of total soluble protein is low in cells with a higher CCM efficiency in chromists, as evidenced by raised *K*_c_ of the Rubisco (Young *et al.*, 2016; Heureux *et al.*, 2017). Low energetic availability (as light or P) tends to reduce CCM activity (Maberly and Gontero, 2017), and interactions with low temperature may also have been significant, whether directly in terms of survival during the Cryogenian snowball earth, or at higher latitudes in terrestrial and marine algae (Raven *et al.*, 2017). One additional driver for a CCM could also occur when inorganic carbon becomes locally depleted within a dense algal bloom, and so may be to some extent independent of equilibration with ambient air (Maberly and Gontero, 2017).

## Convergence in CCM form and function

The three pillars usually invoked to support a CCM (Meyer and Griffiths, 2013) include:

(i)  biophysical inorganic transporters, operating in parallel across adjacent membranes, raising the inorganic carbon pool by some 40-fold (Chlorophyte) to 400-fold (Cyanobacteria) and determining overall affinity and effectiveness of the CCM;
(ii)  a suite of strategically placed carbonic anhydrases (CA) and CA-like moieties, adjacent to the inorganic transporters, to assist in bicarbonate interconversion or regeneration (or recapture) of CO_2_ close to Rubisco;
(iii) a microcompartment within which Rubisco aggregates, and from which CO_2_ leakage is minimized, such as the carboxysome in cyanobacteria and pyrenoid associated with most eukaryotic CCM systems.

Papers in this special issue report on the latest developments in identifying key cyanobacterial and chlorophyte CCM components (Rae *et al.*, 2017), albeit in the context of their potential for introduction into higher plants to augment productivity (see also Price *et al.*, 2013). Cyanobacterial α- and β-carboxysomes regulate the influx of bicarbonate and other metabolite exchanges via pore structures in the proteinaceous shell, with CO_2_ converted internally by CA systems (for details see Rae *et al.*, 2017). The detailed variations between the two cyanobacterial lineages were described by Price *et al.* (2008), but in α-carboxysomes, Form-1A Rubisco is attached to the highly disordered CsoS2 protein, and in β-carboxysomes Form IB Rubisco is integrated via small subunit substitutions to the full-length CcmM protein in an ordered array (Rae *et al.*, 2017).

Additional insights for the carboxysome shell proteins are provided by Sommer *et al.* (2017), who have undertaken a bioinformatic survey of β-carboxysome shell proteins, which suggest that variations in carboxysome structure allow plasticity in response to changing environmental conditions. Meanwhile, Larsson *et al.* (2017) provide crystallographic structural insights for regulation of metabolite exchange by gating of the CcmP protein in the β-carboxysome shell of *Synechococcus elongatus* PCC7942.

The diversity of CCM systems in most photosynthetic eukaryotics mostly requires all three physiological pillars indicated above, and those few which lack an identifiable pyrenoid show reduced capacity for carbon accumulation (Giordano *et al.*, 2005). In this special issue, the comparative evolution of pyrenoids in chlorophytes and chromists is discussed in terms of the commonalities seen in mode of Rubisco aggregation, usually in association with some specialized thylakoid membrane organization (Meyer *et al.*, 2017), although others may be stalked, and in some dinoflagellates Rubisco aggregation is more transient, forming centrally under circadian control (Nassoury *et al.*, 2001).

For chlorophytes, the CCM in *Chlamydomonas* is the best-defined system from a molecular perspective (Meyer and Griffiths, 2013), with hierarchical models presented for regulatory processes leading to CCM induction (Mitchell *et al.*, 2017). Various mutagenic screens and genetic manipulations have helped to characterize components of the *Chlamydomonas* CCM (Li *et al.*, 2016; Machingura *et al.*, 2017), including recent observations on the protein elements associated with Rubisco aggregation (Mackinder *et al.*, 2016; Mitchell *et al.*, 2017), and a new potential thylakoid bicarbonate transporter (Machingura *et al.*, 2017). The observation that specific elements of Rubisco small subunits (SSU) were integral to the aggregation mechanism (Meyer *et al.*, 2012) has led to insights into the function of a possible linker protein (EPYC1, formerly LCI5: Mackinder *et al.*, 2016), and also now for the hierarchical organization of the pyrenoid (Meyer *et al.*, 2017). The *Chlamydomonas* SSU mutants retain the knotted thylakoid tubules that intersect at the heart of the usual pyrenoid location, with growth and photosynthesis restored under elevated CO_2_ supply (Caspari *et al.*, 2017). Such observations suggest that the pyrenoid-associated starch sheath and additional external regulatory elements (LCIB/C) are dependent upon Rubisco aggregation, and the spatial segregation of PSII (normally excluded from within the pyrenoid matrix) does not compromise overall energetic efficiency (Caspari *et al.*, 2017).

For diatoms, whilst the specific details of CCM processes are less well understood than for *Chlamydomonas*, we have more detailed comparative insights into contrasting CCM systems for a wider range of species across the clade. Here, the four layers of thylakoid membranes, associated with secondary plastid endosymbiosis in the Dinophyceae, offer a range of options for concentrating inorganic carbon. Young and Hopkinson (2017) highlight the contrasting trade-offs which seem to have occurred in terms of investment in Rubisco relative to altered Rubisco kinetic properties for contrasting marine habitats. Matsuda *et al.* (2017; see also Tsuji *et al.*, 2017) outline the contrasting modes of carbon uptake and conversion thought to operate in diatoms, dependent on either diffusive entry of CO_2_ or active transport, with one mechanism supported by the more detailed observations seen for the role of CAH1 in *Nannochloropsis oceanica* (Gee *et al.*, 2017).

Further insights are provided by the co-evolution of inorganic carbon transporters (SLC4) in diatoms, used in combination with contrasting CA species (Shen *et al.*, 2017), and also by the use of an intra-thylakoid CA to regenerate CO_2_ adjacent to the aggregated Rubisco (Tsuji *et al.*, 2017), suggesting convergence with the mechanism also proposed for Chlorophytes (Meyer and Griffiths, 2013).

## From molecular diversity to overcoming ecological adversity

The ecological implications of CCM systems are also addressed from an experimental perspective in a number of papers in this special issue. Evolutionary origins (Raven *et al.*, 2017) are complemented by a more detailed comparison of ecological drivers in marine, freshwater and terrestrial habitats by Maberly and Gontero (2017). This leads to a highly original analysis of competitive interactions between cyanobacterial and chlorophyte cells (Ji *et al.*, 2017; see also the Insight article by Beardall and Raven, 2017). These observations are consistent with notions that CCMs help to overcome adversity, as defined above in terms of nutrient availability or local depletion of inorganic carbon within blooms. Thus, chlorophyte algae endure under low ambient CO_2_ equilibration, relative to cyanobacteria, despite their ‘less effective’ CCM; whilst cyanobacteria may thrive under future elevated CO_2_ conditions (Ji *et al.*, 2017; Beardall and Raven, 2017).

The major contribution made by diatoms to biogeochemical cycles, as reviewed by Young and Hopkinson (2017), is further characterized experimentally by a comparison of the effectiveness of the various inorganic carbon accumulation mechanisms (Clement *et al.*, 2017). And finally, although macrophytes make a relatively small contribution to marine net primary productivity (1 PgC per year: Field *et al.*, 1998), the issue contains papers analyzing the mechanisms of inorganic carbon uptake in seagrasses (Larkum *et al.*, 2017) and Antarctic macrophytes (Iñiguez *et al.*, 2017).

## The future

The papers in this special issue convey a renewed sense of excitement and impetus in the field of aquatic carbon concentrating mechanisms, and include contributions from many young scientists with an astonishing breadth of skills, encompassing structural biology, novel molecular manipulations and bioinformatic approaches which are now augmenting traditional physiological and ecological experimentation. Globally, we may face uncertainty, but the potential for CCM systems to enhance marine carbon sequestration (Heureux *et al.*, 2017; Raven *et al.*, 2017; Young and Hopkinson, 2017) or terrestrial crop productivity (Rae *et al.*, 2017), informed by ongoing cutting-edge research programmes, provide some hope, and much promise, for the future.
